# Establishment of patient-derived gastric cancer xenografts: a useful tool for preclinical evaluation of targeted therapies involving alterations in HER-2, MET and FGFR2 signaling pathways

**DOI:** 10.1186/s12885-017-3177-9

**Published:** 2017-03-14

**Authors:** Haiyong Wang, Jun Lu, Jian Tang, Shitu Chen, Kuifeng He, Xiaoxia Jiang, Weiqin Jiang, Lisong Teng

**Affiliations:** 1 Department of surgical oncology, The First Affiliated Hospital, College of Medicine, Zhejiang University, Hangzhou, 310003 Zhejiang Province China; 2 Department of oncology, Jiaxing First Hospital, Jiaxing, 314000 Zhejiang Province China

**Keywords:** Gastric cancer, Xenograft, cMet, Her2, FGFR, Targeted therapy

## Abstract

**Background:**

Targeted therapies are emerging treatment options for gastric cancer (GC). Patient-derived tumor xenograft(PDX) models of GC closely retain the features of the original clinical cancer, offering a powerful tool for preclinical drug efficacy testing. This study aimed to establish PDX GC models, and explore therapeutics targeting Her2, MET(cMet), and FGFR2, which may assist doctor to select the proper target therapy for selected patients.

**Methods:**

GC tissues from 32 patients were collected and implanted into immuno-deficient mice. Using immunohistochemistry(IHC) and fluorescent in-situ hybridization (FISH), protein levels and/or gene amplification of Her2, cMet and FGFR2 in those tissues were assessed. Finally, anti-tumor efficacy was tested in the PDX models using targeted inhibitors.

**Results:**

A total of 9 passable PDX models were successfully established from 32 gastric cancer xenograft donors, consisting of HER2,cMet and FGFR2 alterations with percentages of 4(12.5%), 8(25.0%) and 1(3.1%) respectively. Crizotinib and AZD4547 exerted marked antitumor effects exclusively in PDX models with cMet (G30,G31) and FGFR2(G03) amplification. Interestingly, synergistic antitumor activity was observed in G03 (FGFR2-amplifed and cMet non-amplified but IHC [2+]) with simultaneous treatment with Crizotinib and ADZ4547 at day 30 post-treatment. Further in vitro biochemistry study showed a synergistic inhibition of the MAPK/ERK pathway. HER2,cMet and FGFR2 alterations were found in 17 (10.4%), 32(19.6%) and 6(3.7%) in a group of 163 GC patients, and cMet gene amplification or protein overexpression(IHC 3+) was associated with poor prognosis.

**Conclusions:**

These PDX GC models provide an ideal platform for drug screening and evaluation. GC patients with positive cMet or FGFR2 gene amplification may potentially benefit from cMet or FGFR2 targeted therapies or combined targeted therapy.

**Electronic supplementary material:**

The online version of this article (doi:10.1186/s12885-017-3177-9) contains supplementary material, which is available to authorized users.

## Background

Gastric cancer (GC) is one of the most commonly diagnosed cancers and one of the leading cause of cancer related deaths worldwide [1, 2]. Despite improvements in surgery and chemotherapy, the prognosis of advanced gastric cancer remains poor, with a five-year survival rate of nearly 20% [3]. Over the past decade, targeted therapies have greatly improved the outcomes of patients with certain malignancies, including breast, colorectal, and lung cancer, however, less progress has been made with regard to gastric cancer [4–7]. Therefore, developing new therapeutic approaches, particularly through the use of targeted therapeutic agents, is crucial in gastric cancer research.

One of the main obstacles that hamper progress in therapeutic approaches is the lack of appropriate preclinical models. Conventional cell-implanted xenograft models are commonly used for the development of new drugs. However, prolonged in vitro culture and possible selection cause cell-implanted xenograft models to lose the original molecular characteristics and heterogeneity of primary tumors, which results in poor prediction of the clinical tumor’s drug response [8]. In contrast to cell line–derived xenografts, patient derived tumor xenografts(PDX) closely retain the histopathologic, genetic, and phenotypic features of the patients’ original tumors [8–10]. Recently, PDX models have been widely established for certain tumors, including lung, colorectal, breast, pancreatic, and gastric cancers [9, 11–14]. PDX models are now becoming a powerful tool for the study of tumor biology and the evaluation of anticancer drugs.

Approving trastuzumab for HER2-positive GC patients represents a milestone in targeted therapy for gastric cancer [15]. Recently, ramucirumab(anti-VEGFR2 monoclonal antibody) has been approved for advanced gastric cancer as second-line treatment; however, the improved overall survival by targeted therapy is still limited [16, 17]. Therefore, developing targeted therapeutic agents and increasing the population benefiting from them is urgent in gastric cancer research. MET(cMet) is a member of the RTK family and plays a key role in tumor survival, growth, angiogenesis, and metastasis [18]. A significant proportion of gastric cancers display cMet overexpression and/or gene amplification, and aberrant signaling of cMet pathways in gastric cancer is correlated with advanced tumor stage and poor prognosis [19]. The initial results of preclinical and clinical studies assessing cMet inhibitors such as onartuzumab and crizotinib were negative [20, 21]. FGFR2 is another member of the RTK family that regulates cellular proliferation, survival, migration and differentiation [22].

Approximately 4-7% of gastric cancers show FGFR2 amplification, which may correlate with poor prognosis of gastric cancer patients [23, 24]. A recent study revealed that AZD4547(a selective FGFR kinase inhibitor) exerts marked antitumor effects on GC xenografts carrying FGFR2 gene amplification [25]. Thus, it has become increasingly apparent that FGFR2 is a potential therapeutic candidate for gastric cancer.

In this study, we successfully established nine PDX models using thirty-two implanted GC samples from patients. Then, Her2, cMet, and FGFR2 gene copy number and protein expression levels were assessed in a cohort of 163 GC patients as well as in the 32 GC patients who donated the xenografts. Finally, targeted therapy’s antitumor efficacy was evaluated in PDX models.

## Methods

### Patients and tumor samples

Thirty-two tumor specimens were obtained at initial surgery from 32 gastric patients at the Department of Surgical Oncology, The First Affiliated Hospital, School of Medicine, Zhejiang University. Written informed consent was obtained from each patient, and the study was approved by the hospital ethics committee. This study also included a cohort of 163 patients with GC who received a surgical resection of primary gastric cancer from January 2010 to December 2011 at the Department of Surgical Oncology, The First Affiliated Hospital, School of Medicine, Zhejiang University. The only criteria used for patient selection included the availability of tumor tissue from primary gastric cancer and that of survival data. Follow-up data were obtained by phone, letter, and from the out-patient clinical database, after written informed consent forms provided by all patients. This retrospective study was approved by the institutional review board of the First Affiliated Hospital of Zhejiang University.

### Cell lines and cell culture

The gastric cancer cell lines AGS, KATOIII, SNU5 were purchased from Shanghai Institute for Biological Sciences, Chinese Academy of Science in Sep 2016. The cell lines were characterized by the provider using short tandem repeat (STR) markers. All the cell lines used in this manuscript were tested for mycoplasma contamination in Oct 2016 before setting up for the biochemistry study. Primary GC cells were derived from the tumor excised from the PDX model of G03. Briefly, primary cells were purified with differential adhesion technique and grew in RPMI-1640 medium with 20% fetal bovine serum.

### Reagents

Anti-cMet(ab51067) and anti-Her2(ab134182) antibodies were from Abcam (Cambridge, UK), p-Met antibody (Tyr 1365) (sc-3408), p-ALK antibody (Tyr 1586) (sc-109905), p-Akt1/2/3 antibody (Ser 473)-R (sc-7985-R) and horseradish peroxidase-conjugated secondary antibodieswere purchased from Santa Cruz Biotechnology, Inc. (Santa Cruz, USA). Phospho-FGFR (tyr653/654,#3471), phosphor-p44/p42 MAPK (Erk1/2, #4370), total-Erk1/2 (#4960) were purchased from Cell Signaling Technology. Trastuzumab was obtained from Roche, Inc. (Roche, USA), while crizotinib and ADZ4547 were from Selleck Chemicals, LLC (Houston, CA, USA).

### Cell treatment and Western-blotting

GC cells were seeded at a density of 3 × 10^5^ cells/mL in RPMI-1640 medium containing 10% FBS and cultured overnight. The cells were then incubated with 200nM/L crizotinib or 30 nmol/L AZD4547 for 1 hour or with a combo of both reagent for one hour before being lysed in RIPA cell lysis buffer containing phosphatase and protease inhibitors(sigma). Each 20 μg of protein was loaded onto SDS-PAGE gel; followed by electrophoresis and transferred to polyvinylidene difluoride (PVDF) membranes and probed with antibodies.

### Xenograft models and treatment protocol

Four-to-six-week-old female BALB/c nude mice, purchased from Shanghai Slac Laboratory Animal Corporation (Shanghai, China), were housed with regular 12/12-hour light-dark cycle for at least three days before the study. Animal care was carried out in accordance with the Principles of Laboratory Animal Care (NIH publication#85-23, revised in 1985). All experimental protocols were approved by the Institutional Animal Care and Use Committee of Zhejiang University (approval ID: SYXK[ZHE]2005-0072). Tumor specimens were obtained at initial surgery from patients after written informed consent as mentioned above. PDX gastric carcinoma xenograft models were established as previously described [8, 12]. The tumors were subcutaneously implanted into the flanks of mice under anesthesia with isoflurane; xenograft growth was monitored at least twice weekly by Vernier caliper measuring of the tumor length (a) and width (b).At about 1500 mm^3^, tumors were extracted for serial transplantation. Numerous samples from early passages were stored in the tissue bank, cryopreserved in liquid nitrogen, and used for further experiments. Third generation xenografts (i.e. the second mouse-to-mouse passage) were used for experiments at tumor volumes of about 100-200 mm^3^. Totally, 136 mice were used in this research. Mice with third generation xenografts were randomized divided into several groups (n = 5), including i) vehicle (DMSO as vehicle); ii) AZD4547, daily 6.25 mg/kg oral administration; iii) crizotinib, daily 50 mg/kg oral administration; iv) trastuzumab, weekly 20 mg/kg intraperitoneal injection; v) daily 6.25 mg/kg AZD4547 + 50 mg/kg crizotinib *per o*s. All treatments were administered for 4 weeks, and the dosages were selected according to previous reports [21, 25, 26]. Mouse weights and tumor volumes were assessed daily, with tumor volume derived as (length × width 2)/2. Relative tumor growth inhibition (TGI) was determined by the following formula: TGI = 1 - T/C, where T/C represents the relative tumor growth of compound-treated mice divided by that of control mice. For tumor regression, in which the tumor volume after treatment was smaller than the initial value before dosing, the following equation was used: regression % = 100 × (T0-Ti)/T0. T0 and Ti are tumor volumes in the same group, measured at different time points, with T0 representing the tumor volume on the day preceding the first treatment and Ti the tumor volume at the last measurement day after treatment.

### Immunohistochemistry (IHC)

For immunohistochemical staining, four-micrometer sections were obtained, dewaxed, rehydrated, and subjected to antigen retrieval. After quenching endogenous peroxidase activity and blocking nonspecific binding sites, the sections were incubated with primary antibodies against HER2 (1:100) and cMet (1:100) at 4 °C for 12 h. This was followed by a 30-min incubation with secondary antibodies. Detection was carried out using the streptavidin-biotin peroxidase complex method (LabVision, Fremont, CA, USA) and sections were analyzed under an optical microscope (Nikon, Tokyo Japan; 200×).Her2and cMet expression levels were graded according to Hercep Test guidelines as follows: score 0, no membrane staining or membrane staining in <10% of tumor cells; score 1+, faint/barely perceptible partial membrane staining in >10% of tumor cells; score 2+, weak-to-moderate staining of the entire membrane in >10%of tumor cells; score 3+, strong staining of the entire membrane in >10% of tumor cells. Scores of 0 and1+ were considered negative for overexpression, and scores of 2+ and3+ considered positive. HER2 positive cases were defined by IHC 3+ or IHC 2+ plus Her2 amplification [15]. MET overexpression cases were defined by IHC 2+/3 + .All immunohistochemical slides were reviewed by two independent pathologists.

### Fluorescence in situ hybridization (FISH)

FISH was performed for HER2 and MET gene assessment on 4 μm dewaxed and dehydrated FFPE sections using the HER2/CEN 17 Dual Color Probe (ZytoLight, Cat#Z-2020-20), MET/CEN 7 Dual Color Probe(ZytoLight, Cat# Z-2087-200) and FGFR2/CEN10 Dual Color Probe (ZytoLight, Cat# Z-2122-200) kits, according to the manufacturer's instructions. Probes were co-denatured for 5 min at 80 °C on the slide and incubated overnight at 37 °C. Then, slides were washed with post-hybridization wash buffer(0.5X SSC / 0.1% SDS) for 5 min at 37 °C, air-dried, and counterstained with DAPI dissolved in an anti-fade mounting solution. Using a fluorescence microscope equipped with appropriate filters, the hybridization signals of labeled HER2 /cMet/FGFR2 genes appeared green; those of CEN 17/ CEN 7/ CEN10 appeared orange. The HER2 and cMet genes were considered amplified at FISH signal ratios of HER2/CEP17 or cMet/CEN 7 of ≥2.0 [15, 24, 27]. FGFR2gene amplification was defined as FGFR2/CEP10 ratio ≥2 or tight *FGFR2* gene clusters in ≥10% of the nuclei analyzed per tissue section [25].

### Statistical analysis

Overall survival was measured from the surgery date to death. The Kaplan–Meier method was used to estimate survival distributions, the log-rank test to compare survival distributions, and the Pearson’s chi-squared test or Fisher’s exact test to assess differences between groups. Tumor volume differences between groups were assessed using two-tailed Student’s t-test or one-way ANOVA. *P* < 0.05 was considered statistically significant. Statistical analyses were performed using the SPSS 16.0 software (SPSS, USA).

## Results and discussion

### Results

#### Patient characteristics and establishment of PDX models

**Fig. 1:**
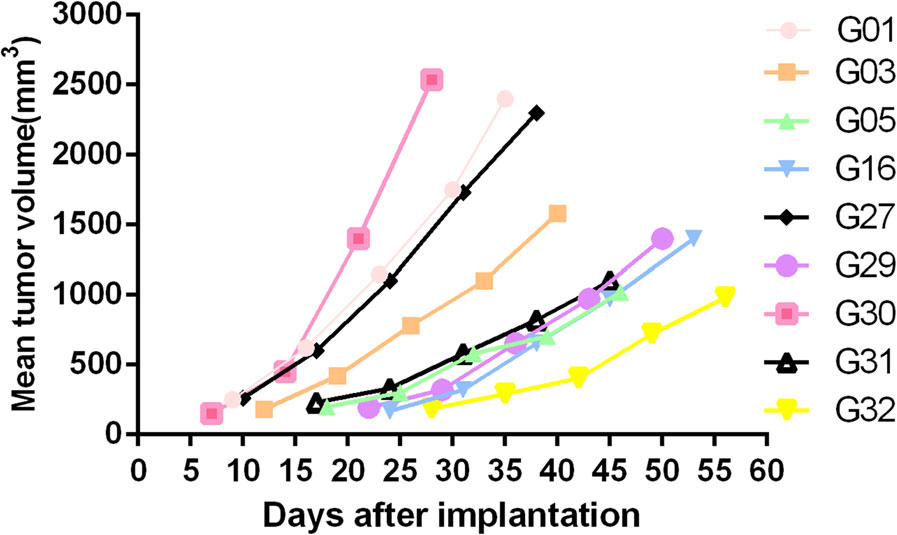
Tumor growth curves of the 9 PDX models at the third generation. The models were established subcutaneously and median tumor volumes in 5 tumor-bearing mice from each model are represented

Nine passable PDX models were established by implantation of GC specimens from 32 patients into immuno-deficient mice. Model passage success rates of first, second, and third generations were 43.7%(14/32),37.5%(12/32), and 28.1%(9/32), respectively. The detailed characteristics of the patients are shown in Table 1. No differences were observed in success rates of model establishment and patient characteristics for different genders, age groups, tumor stages, differentiation statuses, Lauren classes and pre-surgery chemotherapy administration, as shown in Table 1. The established PDX models showed different latency periods (from the day of implantation to palpable tumor) and tumor growth curves (Fig. 1).

**Table 1:** Characteristics of GC patients who donated xenografts for the PDX models

Characteristics	No. of patients (%)	Success rate(≥3 generation)	^a^ *P* value
Gender			0.655
Male	24(75.0%)	6(25%)	
Female	8(25.0%)	3(37.5%)	
Age (years)			0.427
≥60	19(59.4%)	4(26.3%)	
<60	13(40.6%)	5(30.7%)	
Stage			0.648
I/II	6(18.8%)	1(16.7%)	
III/IV	26(81.2%)	8(30.8%)	
Differentiation			0.531
Moderate	8(25.0%)	2(25.0%)	
Moderate-poor	9(28.1%)	4(44.4%)	
Poor and undiff	15(46.9%)	3(20%)	
Lauren classification			0.657
Intestinal	16(50.0%)	5(31.3%)	
Diffuse	11(34.4%)	2(18.1%)	
Mixed	5(15.6%)	2(40%)	
Pre-surgery chemotherapy			0.382
yes	9(28.1%)	1(11.1%)	
no	23(71.9%)	8(34.8%)	

^a^P values are from Fisher’s exact test and were significant at <0.05.undiff,undifferentiated

#### Her2, cMet and FGFR2 status in PDX models and GC patient cohort

**Fig. 2:**
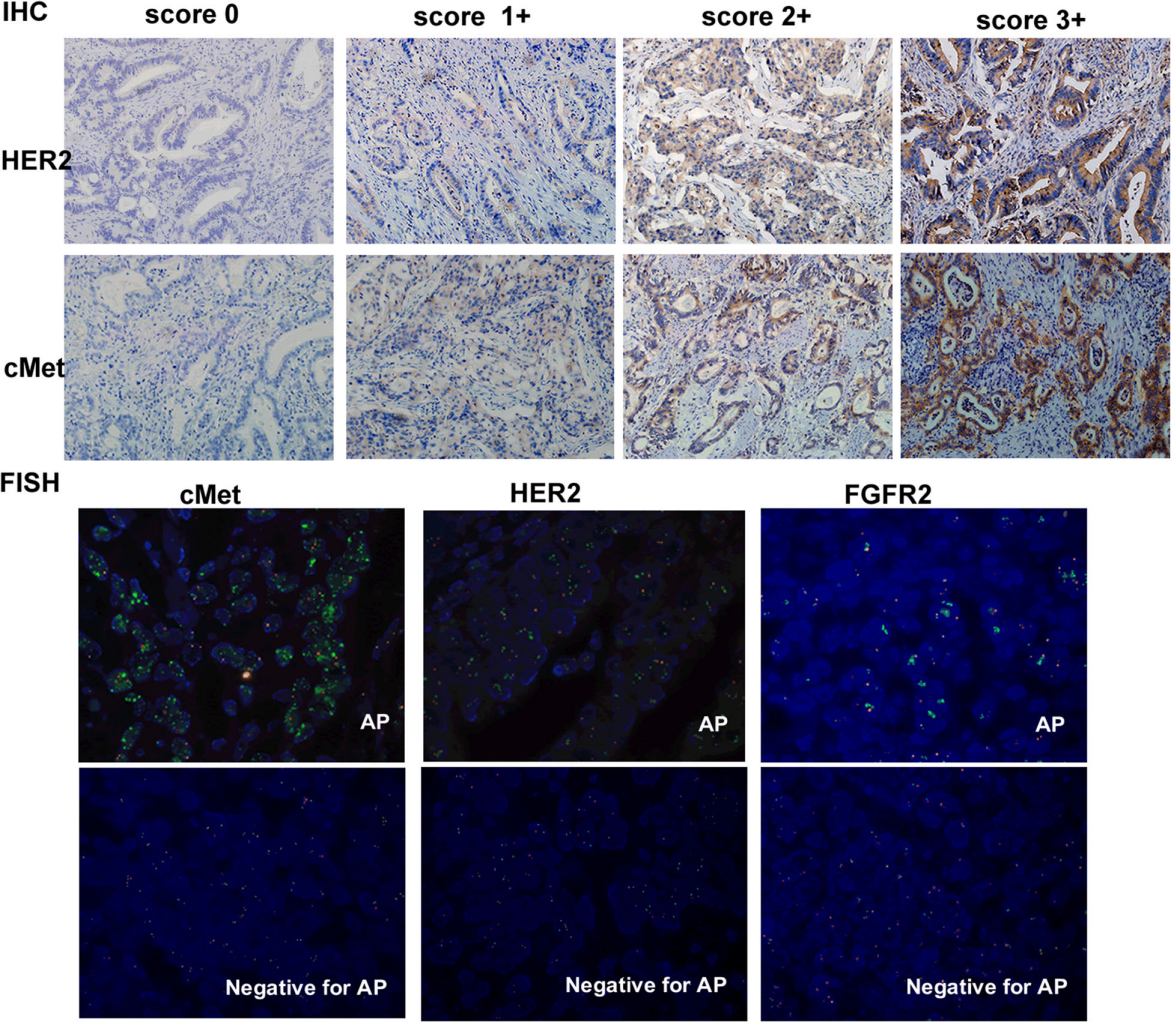
Representative images of IHC and FISH analyses of gastric cancer tumor tissues. Her2 and cMet expression levels were interpreted as scores 0, 1+, 2+, and 3+, respectively. For the FISH assay, orange signals represent Her2,cMet and FGFR2, and the green ones are CEN 17/ CEN 7/ CEN10, respectively. AP, amplification

**Fig. 3:**
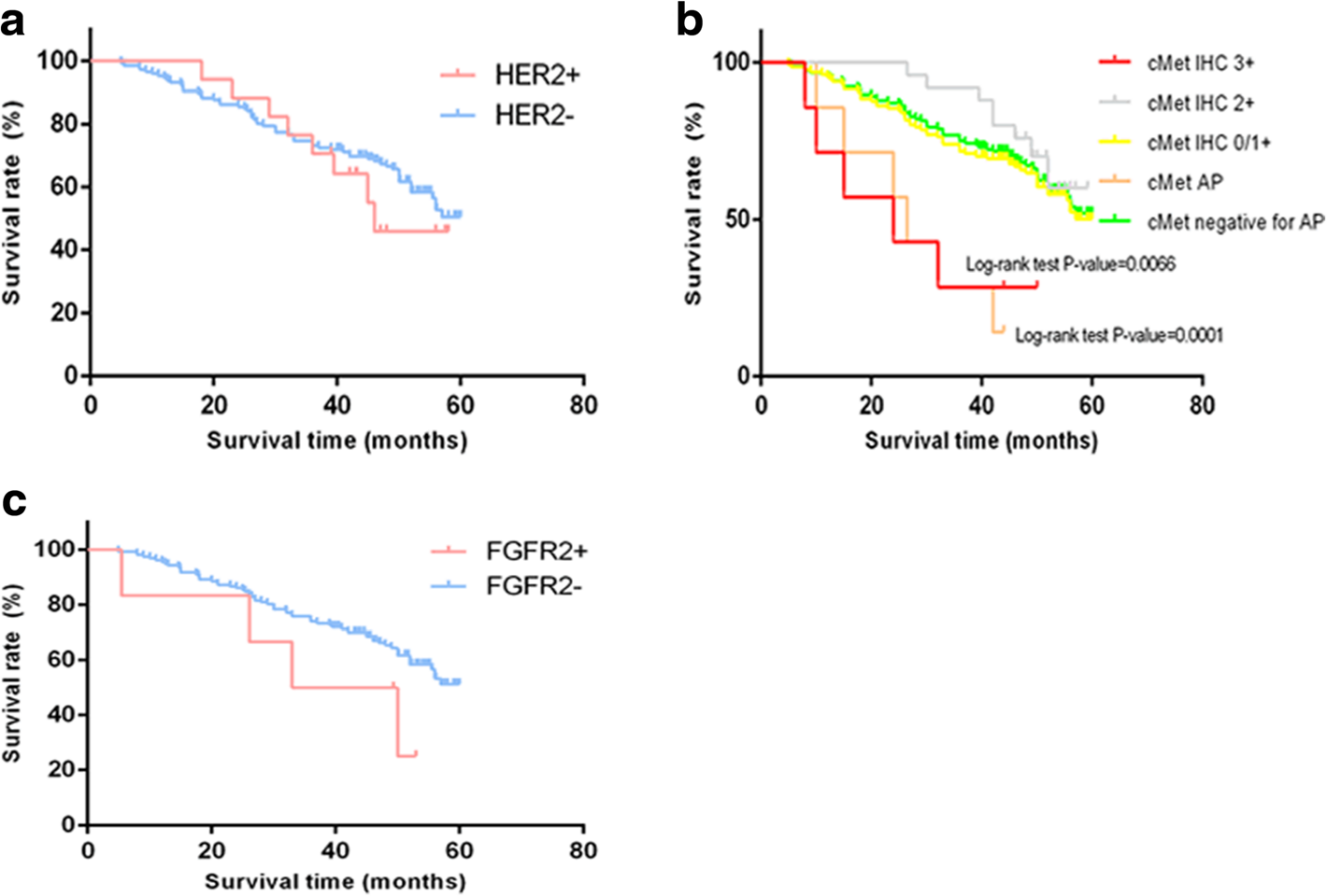
Kaplan-Meier survival analyses of overall survival in a cohort of gastric cancer patients. **a** OS according to Her2 status, Her2+ (IHC3+ or FISH+); **b** OS according to cMet protein expression or gene amplification; **c** OS according to FGFR2 gene amplification. AP, gene amplification

Of the 163 GC patients(detailed characteristics in Additional file 1: Table S1) and 32 GC xenograft donors, HER2 was positive(IHC3+ or FISH +) in 10.4%(17/163)and 12.5%(4/32), respectively; cMet was overexpressed (IHC3+/2+) in 19.6%(32/163) and 25%(8/32), respectively; the cMet gene was amplified in 4.3%(7/163) and 9.4%(3/32), respectively, while FGFR2 gene amplification was found in 3.7%(6/163) and 3.1%(1/32), respectively (Table 2). Representative images of IHC and FISH analyses of gastric cancer tumor tissues are provided in Fig. 2. Patients with cMet protein IHC3+ or gene amplification had poorer survival rates compared with those without IHC3+ or gene amplification (Fig. 3). FGFR2 gene amplification tended to reflect a lower survival rate compared with FGFR2 non-amplificated GC patients, although statistical significance was not reached (Fig. 3). While evaluating Her2, cMet and FGFR2 status in PDX models(≥1 generation, as shown in Table 3), a concordance rate of Her2, cMet and FGFR2 status between primary tumors from patients and PDX models of 92.9% (13/14) was found. Only the G23 model with cMet IHC 3+/FISH(+) changed to cMet IHC 0/FISH(-), as shown in Additional file 2: Figure S1.

**Table 2:** HER2,cMet, and FGFR2 statuses in a cohort of GC patients and corresponding PDX donors

Samples	HER2			cMet				FGFR2
	OP(%)	AP(%)	Positive(%)	OP(%)			AP(%)	AP(%)
	IHC3+	FISH	IHC /FISH	IHC3+	IHC2+	IHC3+/2+	FISH	FISH
A cohort of GC patients (*n* = 163)	12(7.3%)	5(3.1%)	17(10.4%)	7(4.3%)	25(15.3%)	32(19.6%)	7(4.3%)	6(3.7%)
Corresponding patients of PDX models (*n* = 32)	3(9.4%)	1(3.1%)	4(12.5%)	3(9.4%)	5(15.6%)	8(25.0%)	3(9.4%)	1(3.1%)

*OP* protein overexpression, *AP* gene amplification

**Table 3:** Her2,cMet, and FGFR2 statuses of patients and PDX models

	Patients		Patients/PDX models			
Models	Age	Stage	Lauren	Her2	cMet	FGFR2
G01	≥60	III	Mixed	-/-	-/-	-/-
G03	≥60	III	Mixed	-/-	IHC(2+)/FISH(-)	FISH(+)
G05	<60	III	Intestinal	IHC(3+)/FISH(+)	-/-	-/-
G16	<60	III	Intestinal	-/-	-/-	-/-
G27	≥60	III	Diffuse	-/-	-/-	-/-
G29	<60	III	Intestinal	-/-	-/-	-/-
G30	<60	II	Intestinal	-/-	IHC(3+)/FISH(+)	-/-
G31	<60	III	Intestinal	-/-	IHC(2+)/FISH(+)	-/-
G32	≥60	III	Diffuse	-/-	-/-	-/-
G26^a^	≥60	III	Intestinal	IHC(2+)/FISH(-)	-/-	-/-
G28^a^	<60	III	Diffuse	-/-	-/-	-/-
G21^a^	≥60	III	Intestinal	-/-	-/-	-/-
G22^b^	<60	II	Intestinal	-/-	-/-	-/-
G23^b^	≥60	III	Intestinal	-/-	IHC(3+/0)/FISH(+/-)^c^	-/-

^a^PDX models only transplanted to the second generation; ^b^PDX models only transplanted to the first generation; ^c^discordance of cMet status between primary tumors and xenografts in G23

#### Gastric cancer PDX model responses to different molecularly targeted agents

**Fig. 4:**
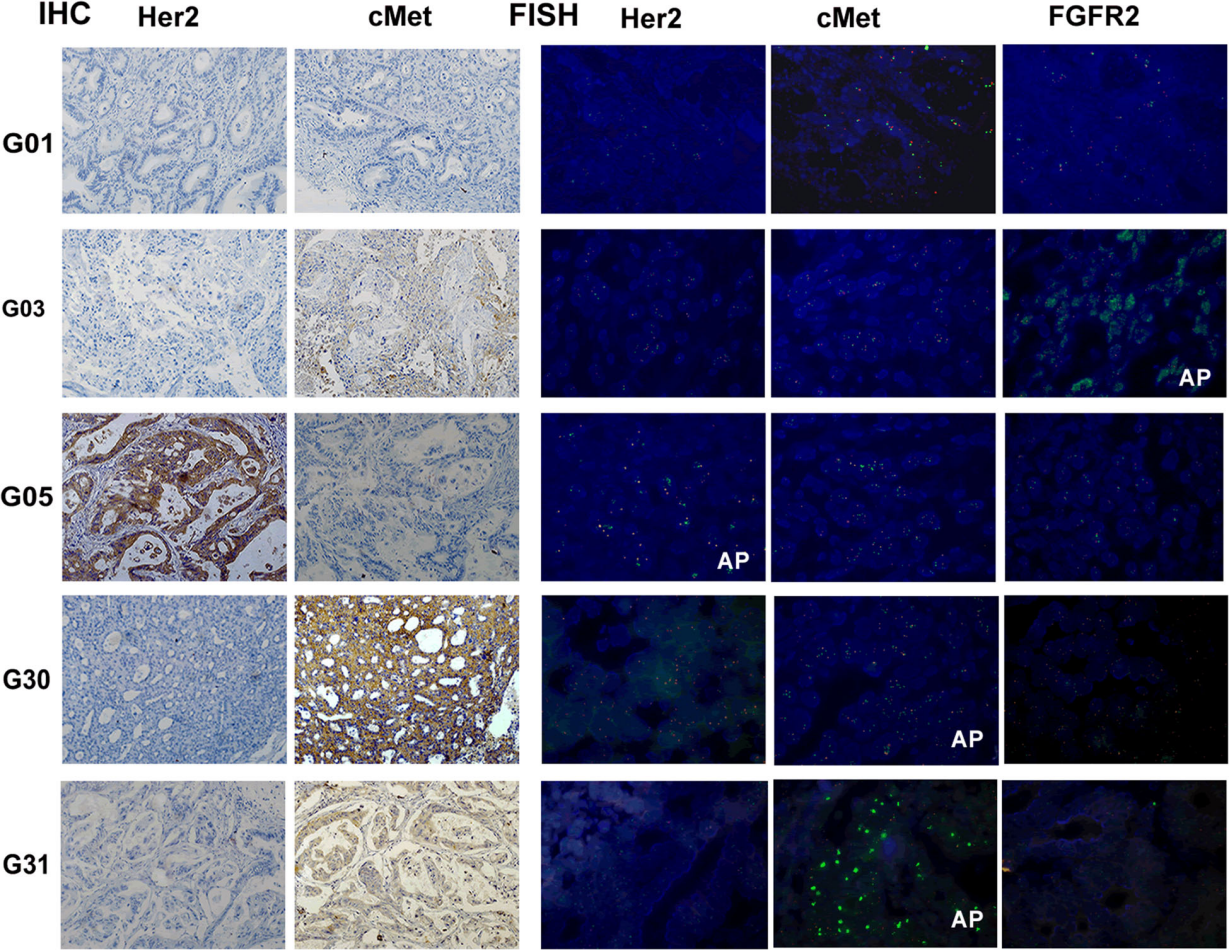
Molecular characterization of the PDX models. Immunohistochemical staining for cMet and HER2, and FISH assay for Her2,cMet and FGFR2 are shown. Immuno-detectable proteins are indicated by brown staining; nuclei are counterstained in blue. Original magnification, ×200

**Fig. 5:**
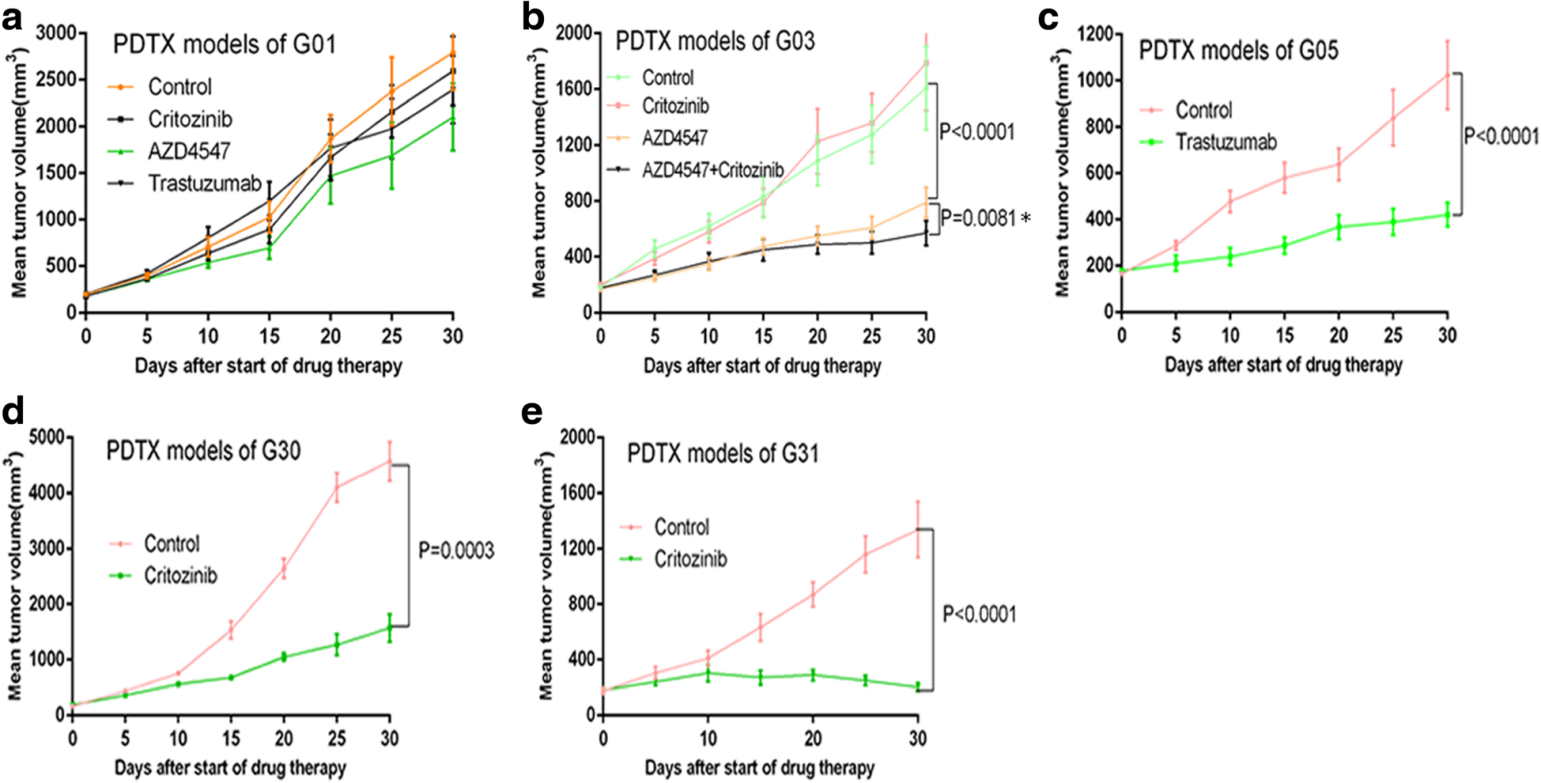
Antitumor efficacy of crizotinib, AZD4547 and trastuzumab in 5 PDX models. Tumor-bearing mice were treated starting from tumor volume of 100 ~ 200 mm^3^. crizotinib, AZD4547, and trastuzumab were administered as described in the experimental section. Subcutaneous tumor volumes were measured using calipers and calculated as mean ± SEM. Statistical analysis of tumor growth inhibition was performed using Student’s *t*-test or one-way ANOVA. P < 0.05 was considered statistically significant. (**a**) Antitumor activity of crizotinib, AZD4547 or Transtuzumab on G01 PDTX models (Her2-, cMet-, FGFR2-); (**b**) Antitumor activity of crizotinib, AZD4547 or combo treatment on G03 PDTX models (FGFR2+); (**c**) Antitumor activity of Transtuzumab on G05 PDTX models (Her2+); (**d**) Antitumor activity of crizotinib on G30 PDTX models (cMet+); (**e**) Antitumor activity of crizotinib on G31 PDTX models (cMet+)

The main purpose of PDX models is to provide a platform for the evaluation of molecularly targeted agents. A total of 9 passable PDX models were established, which harbored alterations of Her2,cMet, and FGFR2(Table 3). Several tyrosine kinase inhibitors which target cMet or FGFR2 pathways show marked antitumor efficacy in gastric cancer; these include crizotinib(cMet TKI)and AZD4547(FGFR2 TKI). cMet or FGFR2 gene amplification seems to be a potential predictive biomarker for drug sensitivity. In order to test the hypothesis that use of crizotinib or AZD4547 could offer therapeutic benefits to GC patients harboring cMet or FGFR2 amplification, PDX models of G30 (cMet-amplified), G31(cMet-amplified), G03 (FGFR2-amplifed and cMet non-amplified but IHC(2+)), G01(both cMet and FGFR2 non-amplified) were assessed in vivo (Fig. 4). Meanwhile, G05(positive for Her2) was also selected for Her2-targeted therapy.We next evaluated the antitumor activity of crizotinib, AZD4547 and trastuzumab on PDX GC models. Crizotinib(50 mg/kg/day), AZD4547 (6.25 mg/kg/day), trastuzumab (20 mg/kg/week) were administered, respectively, for 4 weeks as described above. A significant tumor growth inhibition was observed in G30 and G31(cMet-amplified) after treatment with crizotinib, in contrast to G03 and G01(cMet non-amplified) (Fig. 5a, b, d and e). However, a statistically significant tumor growth inhibition was observed in G03 (FGFR2-amplified)treated with AZD4547, while only minimal to partial tumor growth inhibition was observed in G01 (FGFR2 non-amplified) (Fig. 5a and b). Interestingly, a statistically significant difference in tumor volume was obtained in G03 after treatment with AZD4547 plus crizotinib at day 30 post-treatment (Fig. 5b). Moreover, a marked tumor growth inhibition was observed upon trastuzumab treatment of G05 (Her2-positivy) but not G01 (Her2-negative) (Fig. 5c).

**Fig. 6:**
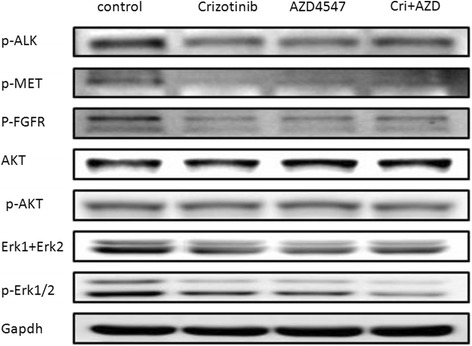
AZD4547 and crizotinib synergistically inhibited activation of MAPK/ERK pathway in G03 xenograft derived GC cells. GC cells derived from G03 xenograft were treated with 200nM/L crizotinib or 30nM/L AZD4547, either alone or as a combo treatment(Cri + AZD) for 1 hour. Whole cell lysates were collected and analyzed by western blot. Cell lysates were immunoblotted for phospho- and total protein as indicated

The gastric cancer cells derived from G03 PDX and commercial gastric cancer cell line AGS, KATOIII, SNU5, were assigned for further biochemistry study. The indicated cells were treated by anti-FGFR2 or anti-MET treatment ,or by a combo treatment of both inhibitors. Total protein was extracted and subsequently sent to Western-blotting study. In the G03 PDX derived cell, which was positive for FGFR2, showed synergetic inhibition of p-ERK expression by a combo treatment of crizotinib and AZD4547, however, no obvious inhibition of AKT was observed (Fig. 6). To determine whether the synergetic effect was cMet or FGFR2 status dependent, we did the same assay in GC cell lines with different status of the two receptors. We also observed the synergetic inhibition of p-ERK expression in the KATOIII cell which was positive of FGFR2 amplification, and in the SNU5 cell which was positive of cMet expression (Additional file 3: Figure S2). However, no synergetic inhibiting effect was observed in AGS cell line which was negative for both receptor expression (Additional file 4: Figure S3).

### Discussion

Mouse xenografts derived from prolonged in vitro cultured cells have been the standard toolkit for cancer biologists for decades; however, the high failure rate of compounds entering clinical trials indicates a low clinical predictive power of traditional tumor model systems.

Compared with cell line derived xenografts, patient derived tumor xenografts models closely retain the histopathologic, genetic and phenotypic features of patients’ original tumors, and could better predict drug efficacy in humans [8–10]. In the last few years, PDX models have been widely established in various tumor, including gastric cancer [9, 11–14]. PDX models are now becoming a valuable tool for evaluating new anticancer drugs before the initiation of clinical trials. The main purpose of this study was to establish and characterize a panel of PDX GC models, mimicking the genetic alteration of gastric cancer patients and further use them as a platform for the evaluation of potential targeted medicine.

Recently, Choi et al. reported the genomic fidelities of the gastric PDX systems and identified factors correlated with engraftment success of PDX tumors. They found the tumor cell percentages in the implanted tissues were correlated with higher success rates [28]. Zhang et al. established 32 PDGCX and the genetic characters of which were confirmed to be consistent with the original tumors [29]. Here, a panel of 9 PDX models were established with a success rate of 28.1%, which is similar to previously reported data [8]. When comparing the success rates between the first, second and third generations, we found the first implantation is a critical step in the process, while the success rate after the third generation nearly reached 100%.No significant correlation was observed between the engraftment achievement ratio and patients’ clinical feature.

However, a recent study revealed that prior chemotherapy may reduce the engraftment achievement ratio [30]. Indeed, patients who showed complete response after pre-surgery chemotherapy were excluded from further implantation in our study. A vital question of the utility of PDX models is whether the passage of tumors in experimental mice alters their histopathological and molecular features. As shown above, only one model with cMet IHC 3+/FISH(+) changed to cMet IHC 0/FISH(-) (Additional file 2: Figure S1); the concordance rate of Her2,cMet and FGFR2 alteration between patients’ primary tumors and xenografts was 92.9% (Table 3). We speculate that tumor heterogeneity may be one of the possible cause of discordance between PDX models and donors. Previous studies have revealed that established PDX models are biologically stable when passaged in mice, in terms of global gene-expression patterns, mutational status, and drug responsiveness [8, 10]. Therefore, PDX models, mimicking the histopathological and molecular features of patients, constitute a superior tool for the preclinical study of new emerging targeted therapies.

Approving trastuzumab for HER2-positive GC patients represents a milestone in gastric cancer targeted therapy [15]. Recently, cMet and FGFR2, two other members of the RTK family, have been intensely investigated in gastric cancer. A high–resolution genomic analysis of a large cohort of gastric primary tumors revealed that approximately 4% and 9% gastric patients harbor cMet and FGFR2 amplification, respectively. The prevalence rates of cMet and FGFR2 alterations in Chinese gastric cancer patients have rarely been reported. Liu et al. reported that nearly 6.1% and 5.1% gastric cancer specimens harbor cMet or FGFR2 amplification in a cohort of Chinese gastric cancer, respectively. Here, we simultaneously studied the status of Her2, cMet and FGFR2 amplification in a cohort of Chinese GC patients and a panel of PDX models. Our data showed cMet overexpression and amplification in 19.6% and 4.3% of GC patients, respectively. Survival analysis revealed that patients with cMet protein IHC3+ or gene amplification have a poorer survival rate, in agreement with a recent study [24]. In addition, FGFR2 amplification was harboured in 3.7% GC patients. FGFR2 amplification tended to indicate a lower survival rate, although a statistical significance was not obtained. A recent international multi-center study reported that FGFR2 amplification is associated with poor prognosis in gastric cancer. Her2 positive rate was 10.4%, which was consistent with previously reported data [31].

Based on the above findings, we confirm that a significant proportion of gastric cancer patients harbour cMet and FGFR2 alterations, and may benefit from cMet and FGFR2 targeted therapies. A phase I study showed that patients with cMet-amplified gastroesophageal cancer treated with crizotinib experience significant tumor shrinkage [32]. In addition, a preclinical study revealed that AZD4547 exerts marked antitumor effects on GC xenografts carrying FGFR2 gene amplification [25]. Therefore, cMet or FGFR2 gene amplification seems to be a potential predictive biomarker for drug sensitivity; however, there is a possible correlation between gene amplification and protein expression as well as possible inconsistencies between IHC data and gene amplifications in terms of tumor take rates, growth kinetics and/or sensitivities to the respective treatment regimens. To further evaluate the translational significance of the above findings, the antitumor efficacy of crizotinib and AZD4547 was assessed on the panel of PDX models generated. As shown above, PDX models with cMet and FGFR2 amplification were highly sensitive to crizotinib, while others showed minimal or even no response. Interestingly, we found that combined treatment of crizotinib and AZD4547 enhances the antitumor effect of AZD4547 in G03 (FGFR2-amplifed and cMet non-amplified but IHC(2+)). The in vitro data showed obvious inhibition of Erk activation by crizotinib in the G03 derived cells, however, crizotinib monotherapy in vivo showed no significant tumor growth inhibition compared with the control group. There might be some mechanism causing this discrepancy which need further investigation. A recent study also demonstrated that activation of several receptor tyrosine kinases (RTKs), including EGFR, HER3 and MET, contributes to AZD4547 hyposensitivity in *FGFR2* amplified GC cells, and the rescue effect was abrogated by inhibiting these RTKs with their targeted tyrosine kinase inhibitors (TKIs) [33]. Another study demonstrated that FGFR is one of the combinatorial targets to overcome resistance to cMet-targeted therapy in gastric cancer [34]. The underlying mechanisms for the enhanced antitumor effect by combined treatment of crizotinib and AZD4547 in G03 is still unknown. By using the G03 xenograft derived cells, in vitro assay showed that a combination treatment of crizotinib and AZD4547 led to synergetic inhibition of MAPK/ERK pathway. Further biochemistry study on the GC cell lines with different status of cMet or FGFR2 amplification showed that the synergetic effect were obtained only in cMet or FGFR2 amplified cells, we speculated that co-targeting cMet and FGFR2 may exhibit a synergetic tumor inhibition through MAPK/ERK pathway. We observed the trans-phosphorylation of MET and FGFR2, however, the trans-phosphorylation were not consistent in the four cell lines(data not shown). The synergistic effect of the combo treatment of the crizotinib and FGFR2 inhibitor at the level of ERK phosphorylation is consistent in all the four different cell lines except the AGS cells which is negative for both receptor expression. We believe that the molecular mechanism underlying the synergistic effect of concomitant inhibition of the two parallel pathways, is more like to involve the downstream effectors of MET and FGFR2, but not the transphosphorylation of the two parallel receptors. Further studies are needed to explore the crosstalk between cMet and FGFR2 signaling pathway. Co-targeting cMet and FGFR2 may be a promising strategy for gastric cancer patients with amplification of cMet or FGFR2.

## Conclusions

In conclusion, a panel of 9 PDX GC models were successfully established, providing an ideal platform for the evaluation of targeted agents. In addition, Her2, cMet and FGFR2 statuses were profiled in a cohort of GC patients and the PDX models. Finally, our data indicate that a significant proportion of GC patients harbouring cMet or FGFR2 gene amplification can benefit from cMet or FGFR2 targeted therapies or combined targeted therapies.

## Additional files

Additional file 1: Table S1. Characteristics of GC cohort patients. The clinical and pathological characteristics of 163 GC cohort patients were described in the table. (DOC 34 kb)
Additional file 2: Figure S1. Discordance of cMet status between primary tumors and xenografts in G23. cMet status of the primary tumor and first generation of G23 model were analyzed by IHC and FISH, results showed the discordance between primary tumors and xenografts. (DOC 277 kb)
Additional file 3: Figure S2. MAPK/ERK pathway was inhibited synergistically by a combination of crizotinib and AZD4547 in MET or FGFR2 amplified GC cells. GC cell line KATOIII(FGFR2 amplified) or SNU05(cMet amplified) was treated with 200nM/L crizotinib or 30nM/L AZD4547, either alone or as a combo treatment(Cri + AZD) for 1 hour. Cell lysates were immunoblotted for phospho- and total ERK1/2. (DOC 177 kb)
Additional file 4: Figure S3. No synergetic inhibition of ERK activation was observed in AGS cell line which was negative for MET or FGFR2 expression. (DOC 90 kb)

## Abbreviations

FGFR: Fibroblast growth factor receptor
FISH: Fluorescence in situ hybridization
GC: Gastric cancer
IHC: Immunohistochemistry
PDX: Patient derived tumor xenografts
RTK: Receptor tyrosine kinase
TKIs: Tyrosine kinase inhibitors
