# Supplementary material for: Establishment of patient-derived gastric cancer xenografts: a useful tool for preclinical evaluation of targeted therapies involving alterations in HER-2, MET and FGFR2 signaling pathways
Source: BMC Cancer. 2017 Mar 14;17:191. doi: 10.1186/s12885-017-3177-9 (PMC5348902; doi:10.1186/s12885-017-3177-9)
Supplement: Additional file 1: Table S1. — Characteristics of GC cohort patients. The clinical and pathological characteristics of 163 GC cohort patients were described in the table. (DOC 34 kb) [file 12885_2017_3177_MOESM1_ESM.doc]

| **Table S1.** Characteristics of GC cohort patients. | |
| --- | --- |
| Characteristics | No. of patients (%) |
| Gender |  |
| Male | 118(72.4%) |
| Female | 45(27.6%) |
| Age (years) |  |
| ≥60 | 98(60.1%) |
| <60 | 65(39.9%) |
| TMN Stage |  |
| I | 22(13.5%) |
| II | 49(30.1%) |
| III | 82(50.3%) |
| IV | 10(6.1%) |
| Differentiation |  |
| High | 6(3.7%) |
| Moderate | 31(19.0%) |
| Moderate-poor | 49(30.1%) |
| Poor and undiff | 77(47.2%) |
| Lauren classification |  |
| Intestinal | 78(47.9%) |
| Diffuse | 68(41.7%) |
| Mixed | 17(10.4%) |

The clinical and pathological characteristics of 163 GC cohort patients were described in the table.
