# Supplementary material for: Establishment of patient-derived gastric cancer xenografts: a useful tool for preclinical evaluation of targeted therapies involving alterations in HER-2, MET and FGFR2 signaling pathways
Source: BMC Cancer. 2017 Mar 14;17:191. doi: 10.1186/s12885-017-3177-9 (PMC5348902; doi:10.1186/s12885-017-3177-9)
Supplement: Additional file 2: Figure S1. — Discordance of cMet status between primary tumors and xenografts in G23. cMet status of the primary tumor and first generation of G23 model were analyzed by IHC and FISH, results showed the discordance between primary tumors and xenografts. (DOC 277 kb) [file 12885_2017_3177_MOESM2_ESM.doc]

**Figure S1**. Discordance of cMet status between primary tumors and xenografts in G23.


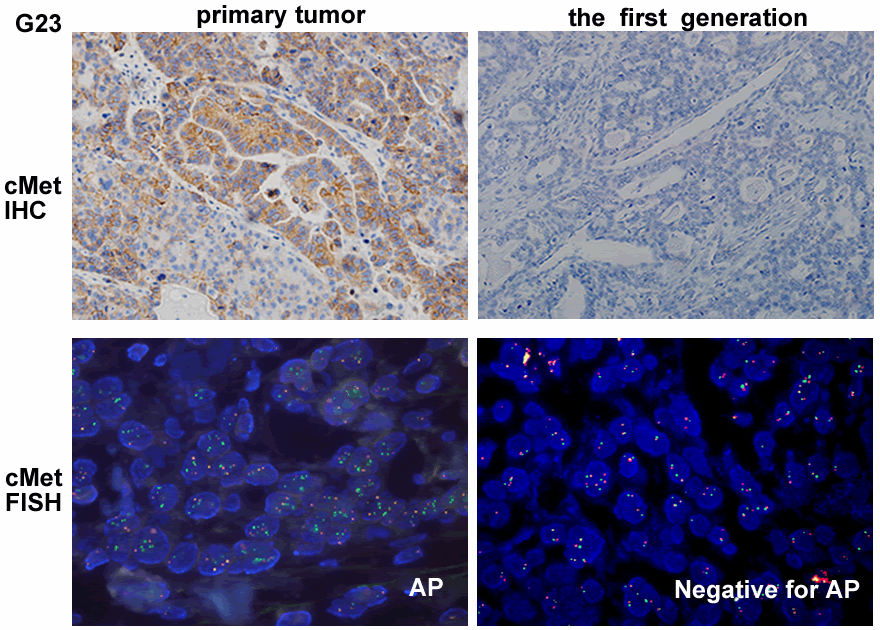


cMet status of the primary tumor and first generation of G23 model were analyzed by IHC and FISH, results showed the discordance between primary tumors and xenografts.
