# Supplementary material for: Establishment of patient-derived gastric cancer xenografts: a useful tool for preclinical evaluation of targeted therapies involving alterations in HER-2, MET and FGFR2 signaling pathways
Source: BMC Cancer. 2017 Mar 14;17:191. doi: 10.1186/s12885-017-3177-9 (PMC5348902; doi:10.1186/s12885-017-3177-9)
Supplement: Additional file 3: Figure S2. — MAPK/ERK pathway was inhibited synergistically by a combination of crizotinib and AZD4547 in MET or FGFR2 amplified GC cells. GC cell line KATOIII(FGFR2 amplified) or SNU05(cMet amplified) was treated with 200nM/L crizotinib or 30nM/L AZD4547, either alone or as a combo treatment(Cri + AZD) for 1 hour. Cell lysates were immunoblotted for phospho- and total ERK1/2. (DOC 177 kb) [file 12885_2017_3177_MOESM3_ESM.doc]

**Figure S2** MAPK/ERK pathway was inhibited synergistically by a combination of crizotinib and AZD4547 in MET or FGFR2 amplified GC cells.
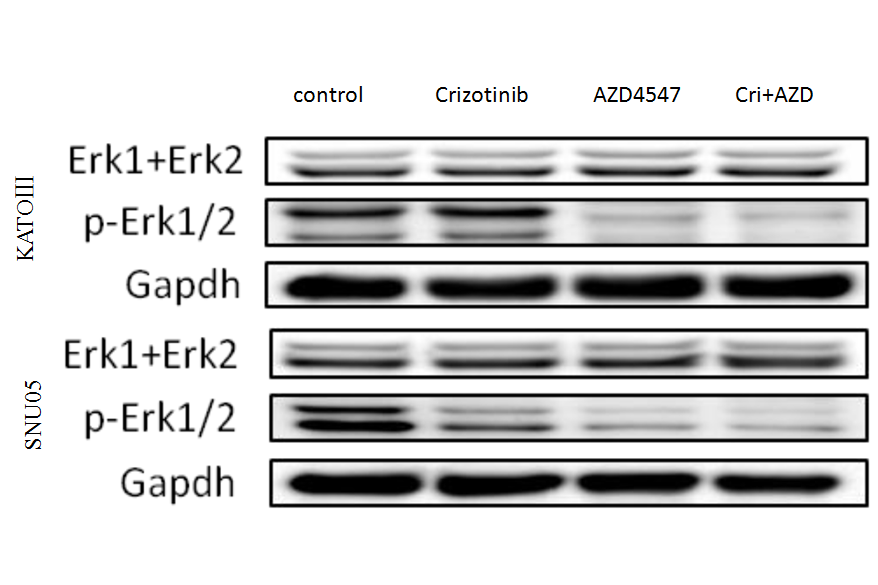


GC cell line KATOⅢ(FGFR2 amplified) or SNU05(cMet amplified) was treated with 200nM/L crizotinib or 30nM/L AZD4547, either alone or as a combo treatment(Cri+AZD) for 1 hour. Cell lysates were immunoblotted for phospho- and total ERK1/2.
