# Supplementary material for: Establishment of patient-derived gastric cancer xenografts: a useful tool for preclinical evaluation of targeted therapies involving alterations in HER-2, MET and FGFR2 signaling pathways
Source: BMC Cancer. 2017 Mar 14;17:191. doi: 10.1186/s12885-017-3177-9 (PMC5348902; doi:10.1186/s12885-017-3177-9)

**Figure S3**. No synergetic inhibition of ERK activation was observed in AGS cell line which was negative for MET or FGFR2 expression.


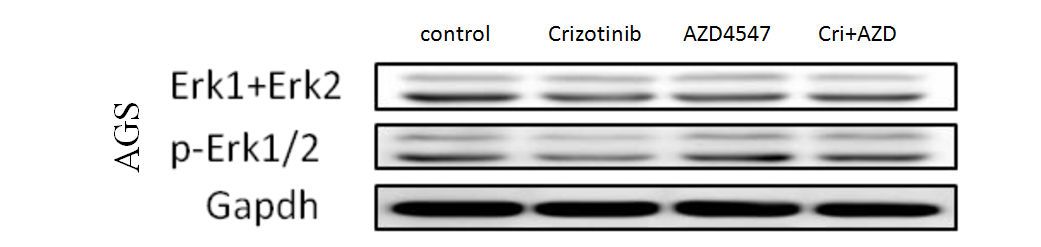

Supplement: Additional file 4: Figure S3. — No synergetic inhibition of ERK activation was observed in AGS cell line which was negative for MET or FGFR2 expression. (DOC 90 kb) [file 12885_2017_3177_MOESM4_ESM.doc]
